# ITRAQ-based quantitative proteomic analysis of *Cynops orientalis* limb regeneration

**DOI:** 10.1186/s12864-017-4125-4

**Published:** 2017-09-22

**Authors:** Jie Tang, Yuan Yu, Hanxue Zheng, Lu Yin, Mei Sun, Wenjun Wang, Jihong Cui, Wenguang Liu, Xin Xie, Fulin Chen

**Affiliations:** 1 Lab of Tissue Engineering, Faculty of Life Science, Northwest University, 229 Taibai North Road, Xi’an, Shaanxi Province 710069 People’s Republic of China; 2 Provincial Key Laboratory of Biotechnology of Shaanxi, Northwest University, 229 Taibai North Road, Xi’an, Shaanxi Province 710069 People’s Republic of China; 3 Key Laboratory of Resource Biology and Modern Biotechnology in Western China, Ministry of Education, Northwest University, 229 Taibai North Road, Xi’an, Shaanxi Province 710069 People’s Republic of China; 4 Shaanxi Institute of Zoology, 88 Xingqing Road, Xi’an, Shaanxi Province 710032 People’s Republic of China

**Keywords:** *Cynops orientalis*, iTRAQ, Proteome, Limb regeneration

## Abstract

**Background:**

Salamanders regenerate their limbs after amputation. However, the molecular mechanism of this unique regeneration remains unclear. In this study, isobaric tags for relative and absolute quantification (iTRAQ) coupled with liquid chromatography tandem mass spectrometry (LC-MS/MS) was employed to quantitatively identify differentially expressed proteins in regenerating limbs 3, 7, 14, 30 and 42 days post amputation (dpa).

**Results:**

Of 2636 proteins detected in total, 253 proteins were differentially expressed during different regeneration stages. Among these proteins, Asporin, Cadherin-13, Keratin, Collagen alpha-1(XI) and Titin were down-regulated. CAPG, Coronin-1A, AnnexinA1, Cathepsin B were up-regulated compared with the control. The identified proteins were further analyzed to obtain information about their expression patterns and functions in limb regeneration. Functional analysis indicated that the differentially expressed proteins were associated with wound healing, immune response, cellular process, metabolism and binding.

**Conclusions:**

This work indicated that significant proteome alternations occurred during salamander limb regeneration. The results may provide fundamental knowledge to understand the mechanism of limb regeneration.

**Electronic supplementary material:**

The online version of this article (10.1186/s12864-017-4125-4) contains supplementary material, which is available to authorized users.

## Background

Some urodele amphibians possess the extraordinary capacity to regenerate their damaged body parts, including limbs and tails [1], jaw [2], spinal cord [3], lens [4, 5], retina [6], neuron [7] and heart [8]. For example, the missing portion of a salamander limb could be perfectly regenerated after amputation between the shoulder and hand.

Salamander limb regeneration could be divided into three major stages. The first stage is the covering of the wound. Epidermal cells around the stump migrate rapidly into the wound area and form an epithelial structure called the wound epidermis (WE) within 1 day after amputation [9]. During the second stage, cells surrounding the injury site begin to dedifferentiate, proliferate and form a mesenchymal growth zone known as the blastema. Simultaneously, the WE thickens apically to form an apical epidermal cap (AEC) [10]. In the last stage, cells in the blastema undergo patterned differentiation to generate diverse tissue structures and replicate the amputated limb parts [11]. The blastema is a heterogeneous collection of mesenchymal progenitor cells originated from mature limb tissues, including the dermis, muscle, cartilage and connective tissue underneath WE. Importantly, cells from specific tissue produce progenitor cells with restricted differentiation potential. The dermis makes epidermis, cartilage and tendons, but not muscle or Schwann cells, and muscle does not make cartilage or epidermis. Furthermore, cartilage-derived blastema cells possess positional identity, whereas Schwann-derived cells do not [11].

Regeneration of limbs is a complex process involving the activation of a number of biological processes, signaling pathways and large-scale tissue remodeling [12–17]. Understanding the mechanisms underlying in limb regeneration offers important insights into the possibilities for regenerating complex structures in adult vertebrates. Great efforts have been made to identify expression profiles and the functional role of specific genes during limb regeneration [18–20].

Strategies of comparative proteome have been employed to reveal substantial changes in the proteome composition and identify critical molecular during salamander limb regeneration. Rao et al. [21] identified 309 proteins that had significant changes relative to controls during blastema formation with label-free liquid chromatography/mass spectrometry. These proteins were involved in various biological process, including signaling, Ca^2+^ binding and translocation, transcription, translation and cell cycle. The expression of ecotropic viral integrative factor 5 (EVI5), a cell cycle-related oncoprotein, was exceptionally high throughout blastema formation. Geng et al. [22] revealed 212 proteins that participated in the differentiation of skin cells, myocytes, neurocytes, chondrocytes and osteocytes during limb regeneration, via two-dimensional fluorescence difference gel electrophoresis (2D–DIGE) and mass spectrum (MS) analyses. The combination of isobaric tagging for relative and absolute protein quantification (iTRAQ), LC-MS/MS and protein Mascot search engine is a powerful method to quantitatively identify differentially expressed proteins (DEPs) with high accuracy and reproducibility [23, 24]. In this study, we employed this method to screen proteome alterations in regenerating limbs 3, 7, 14, 30 and 42dpa. We detected 2636 proteins in total, and 253 proteins were differentially expressed during different regeneration stages. Functional bioinformatics analysis suggested that these proteins involved in various biological process categories, including wound healing and cell differentiation.

## Results

### Gross inspection and histological observation of regenerating limb

**Fig. 1:**
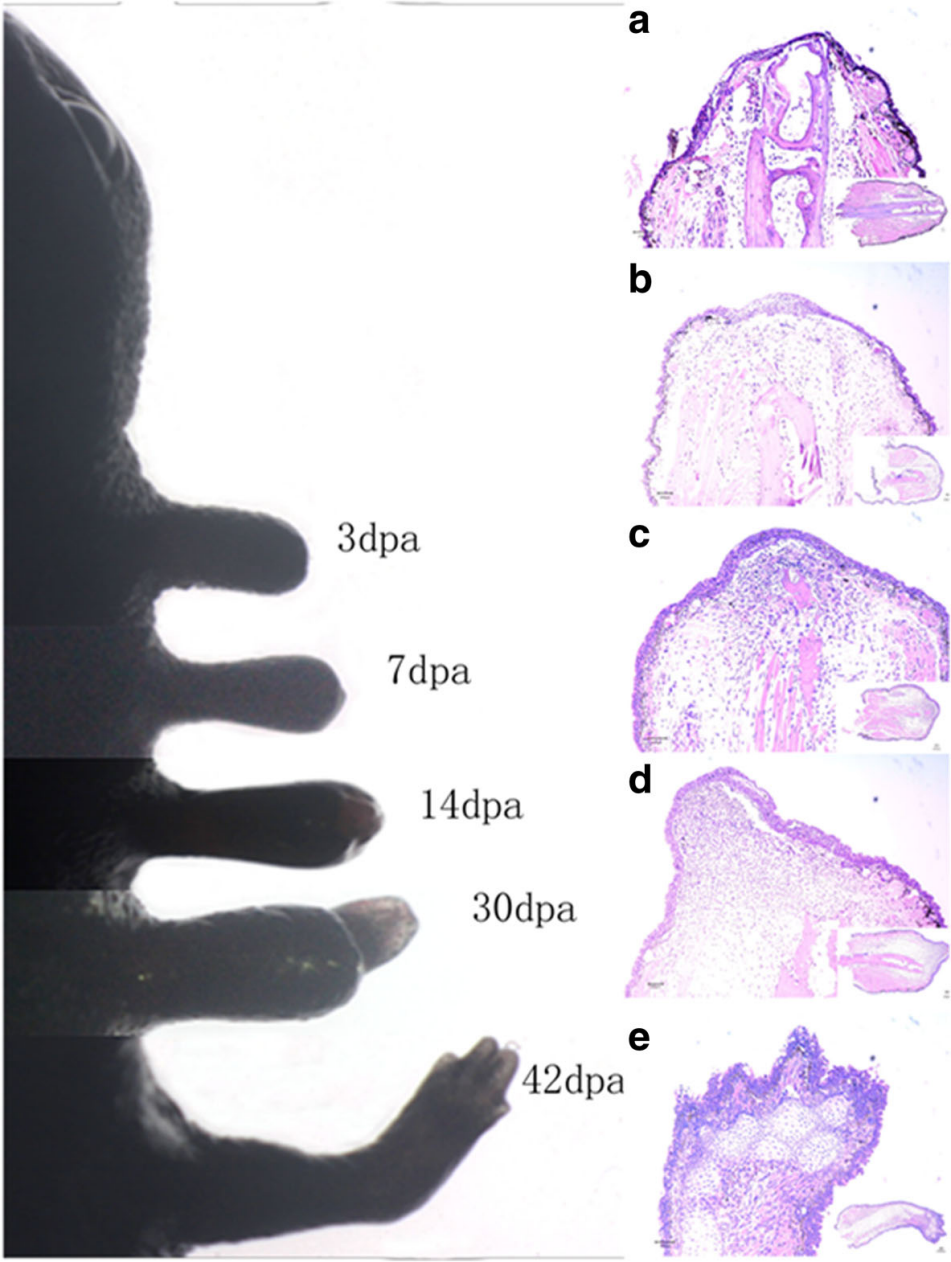
The morphological and histological appearance at different days post amputation. Gross inspection of *Cynops orientalis* limb regeneration (left). Samples were amputated 1 mm over the elbow at different days post-amputation. HE staining of longitudinal sections of regenerating limbs at different days post amputation (right). **a** Section at 3dpa. Wound stump covered by epidermis and gland cells without basement membrane. **b** Section at 7dpa. Undifferentiated mesenchymal cells accumulating under the thickened AEC (M: muscle, B: bone). **c** Section at 14dpa. Formation of blastema. **d** Section at 30dpa. Formation of initial limb bud. **e** Section of 42dpa. Regenerating limb with initial digits

Figure 1 presents the integrated morphological and histological staging scheme of salamander limb regeneration used in the current experiment. Histological observation of regenerating limbs in the longitudinal section showed that the wound stump was covered by epidermis and gland cells. The wound epidermis lacked a basement membrane and directly contacted the underlying mesenchymal tissues at 3dpa. Muscle fragments, cellular debris and lymphocytes could be observed under the WE (Fig. 1a). At 7dpa, the WE thickened and formed an AEC. Cells from the surrounding dermal tissue, muscle (M) and bone (B) dedifferentiated and accumulated under the AEC (Fig. 1b). By 14dpa, the amount of undifferentiated mesenchymal cells increased and formed a blastema (Fig. 1c). By 30dpa, the blastema developed to form an early limb bud (Fig. 1d). At 42dpa, limb with initial digits was regenerated (Fig. 1e).

### Protein identification and quantification

Tryptic peptides were labeled with iTRAQ tags, and the analytical separation and identification of the samples for each biological replicate were performed by LC-MS/MS. A total of 6042 unique peptides were detected, and 2636 proteins with a mascot score of at least 13 were identified between the experimental and control groups. (Additional file 1: Table S1).

### Differentially expressed proteins analysis

**Fig. 2:**
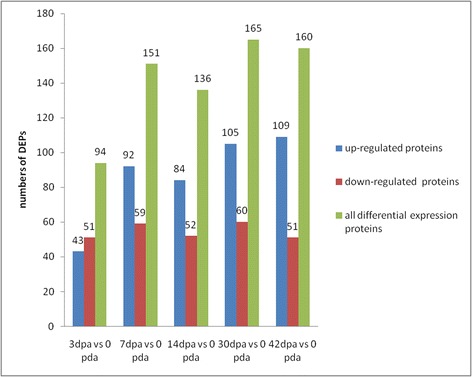
Numbers of DEPs in the regenerated limb tissues compared with the control group

The relative quantification of proteins was based on the ratio of the peak areas from the MS/MS spectra. Among them, 590 proteins had quantitative information. In the current experiment, proteins with iTRAQ ratios below the low range (0.8) were considered to be down-regulated, whereas those above the high range (1.2) were considered to be up-regulated. The identified DEPs were also detected in at least two of the three biological replicates. Based on these criteria, we identified 253 proteins with a significant difference among the regenerating groups compared with the control group (Fig. 2 and Additional file 2: Table S2). Our findings showed that the number of DEPs peaked in the temporal comparison of 30dpa vs. control, 42dpa vs. control, and 7dpa vs. control.

Among these DEPs, 153 DEPs were up-regulated (Additional file 3: Figure S1A, Additional file 2: Table S2) and Myeloperoxidase (MPO) was the most up-regulated protein (3dpa vs. control ratio; mean 5.491). The up-regulated proteins were involved in transport and metabolism, posttranslational modification, transcription, cytoskeleton, such as macrophage-capping protein (CAPG), coronin-1A (CORO1A), annexinA1 (ANXA1), hemopexin, cathepsin B (CTSB), and eukaryotic translation initiation factor 5A isoform II (EIF5A2). In total, 87 DEPs were down-regulated (Additional file 3: Figure S1B, Additional file 2: Table S2) and involved in transcription, signal transduction mechanisms, transport and metabolism. The down-regulated DEPs included extracellular matrix organization-related proteins, such as keratin, type II cytoskeletal, annexinA 2-A(anxa2-a), matrilin-4(MATN4), cadherin-13 (CDH13), basement membrane-specific heparan sulfate proteoglycan core protein (HSPG2), and collagen alpha-1(XI) chain (COL11A1). Keratin was the most down-regulated protein (3dpa vs. control ratio; mean 0.06633). A total of 13 DEPs were identified that exhibited a variation trend as a wave change post amputation between the experimental and control groups.

### Function and bioinformatics analyses

**Fig. 3:**
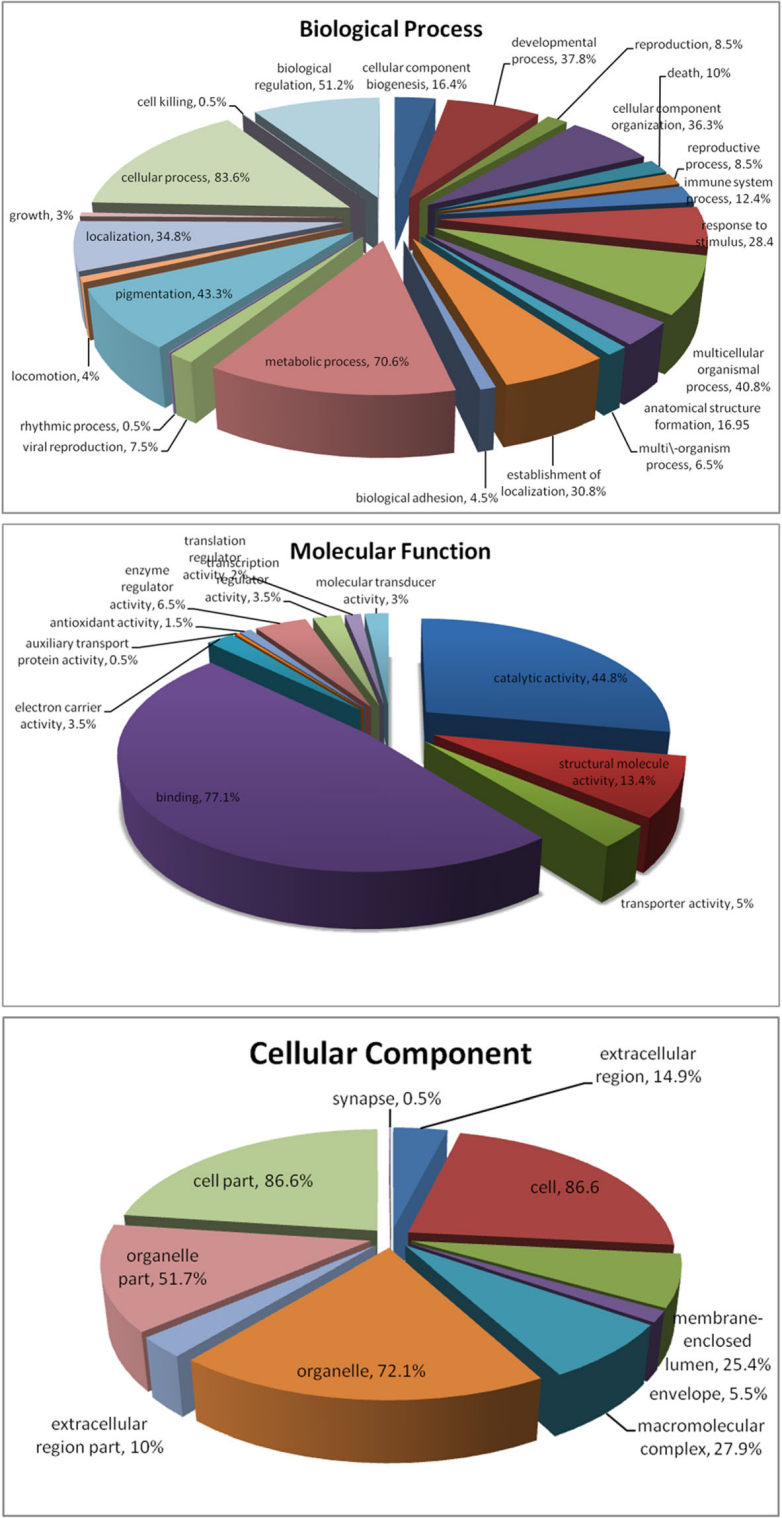
GO distribution analysis of differentially expressed proteins during regeneration from the control and treatment groups (3, 7, 14, 30 and 42dpa). In total, 200 differentially expressed proteins were categorized based on “Cellular Component”, “Molecular Function”, and “Biological Process” using WEGO

Based on Gene Ontological (GO) analysis of DEPs, there’re 200 proteins which have GO annotations and can be categorized functionally using WEGO (Fig. 3). A protein was counted more than once when the protein was assigned to more than one category. Figure 3 stratifies the proteins according to biological process, cellular component and molecular function. The DEPs were grouped into 23 biological process categories: cellular processes (83.6%), metabolic processes (70.6%), biological regulation (51.2%), pigmentation (43.3%), multicellular organismal processes (40.8%), developmental processes (37.8%), cellular component organization (36.3%), localization (34.8%), establishment of localization (30.8%), response to stimulus (28.4%), anatomical structure formation (16.9%), cellular component biogenesis (16.4%), immune system processes (12.4%), death (10.0%), reproduction (8.5%), reproductive process (8.5%), viral reproduction (7.5%), multi-organism process (6.5%), biological adhesion (4.5%), growth (3.0%), locomotion (4.0%), rhythmic process (0.5%) and cell killing (0.5%). Important molecular functions associated with the datasets include binding, catalytic activity, and structural molecule activity. A detailed GO annotation of the differentially expressed proteins was provided in Additional file 4: Table S3.

**Fig. 4:**
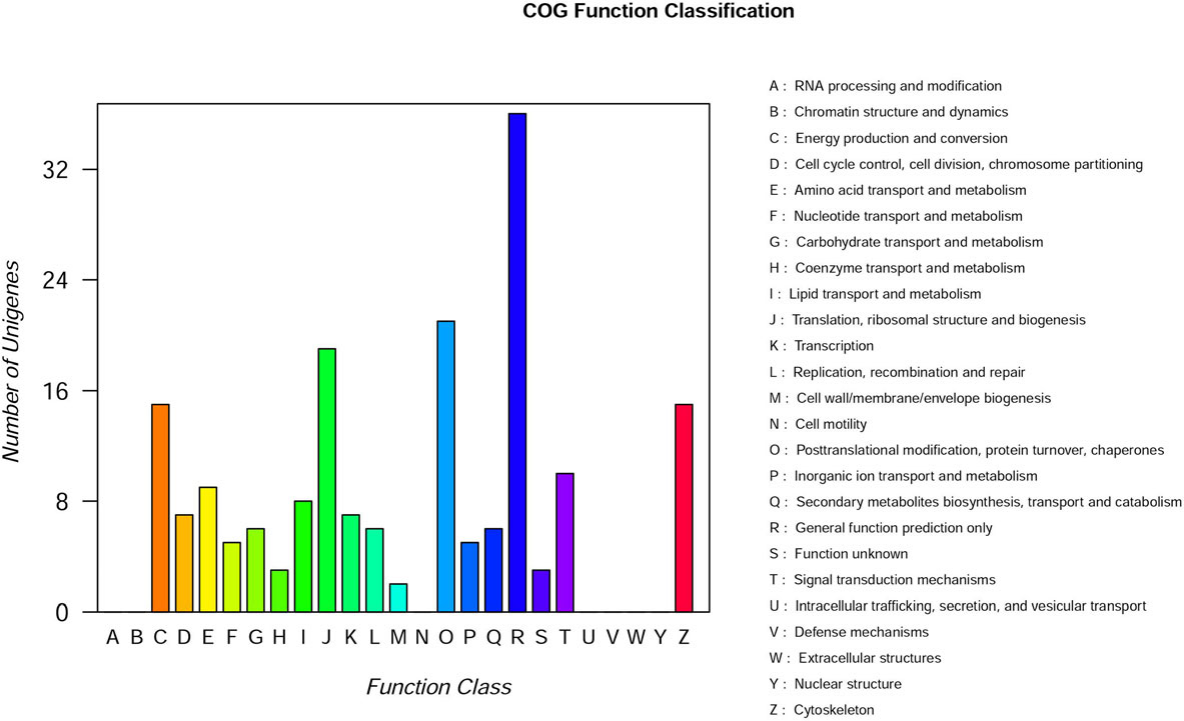
COG classification of differentially expression proteins in iTRAQ data. The X-axis represents the COG categories, whereas the Y-axis represents protein quantity. The picture displays the statistics as the number of different functional proteins in the sample

Those differentially expressed proteins with significant changes were profiled based on their COG functional classification and categorized into as many as 18 COGs (Fig. 4). “General function prediction only” represented the largest group, followed by “Posttranslational modification, protein turnover, chaperones” and “translation, ribosomal structure and biogenesis”.

To identify the biological pathways involved in limb regeneration, we mapped the annotated sequences to the reference canonical pathways in the Kyoto Encyclopedia of Genes and Genomes (KEGG). There’re 232 KEGG pathway annotated by all these DEPs. (Additional file 5: Table S4). When the FDR-corrected *P*-value was set as <0.05, 29 KEGG pathways were identified to be significant (Table 1).

**Table 1:** KEGG pathway enrichment analysis of differential expression proteins

Kegg pathway	Numbers of proteins					*p*-value				
	3 dpa	7 dpa	14 dpa	30 dpa	42 dpa	3 dpa	7 dpa	14 dpa	30dpa	42dpa
ko04512 ECM-receptor interaction	7	/	6	6	/	0.000188	/	0.016531	0.032279	/
ko04974 Protein digestion and absorption	5	6	6	6	/	0.000825	0.001014	0.000898	0.001982	/
ko05150 *Staphylococcus aureus* infection	6	13	7	/	/	0.005453	0.001032	0.014634	/	/
ko05414 Dilated cardiomyopathy	8	13	13	14	15	0.011778	0.001032	0.00083	0.001001	0.000197
ko04145 Phagosome	8	12	16	14	12	0.016209	0.005786	2.12E-05	0.001828	0.01271
ko05410 Hypertrophic cardiomyopathy (HCM)	7	13	12	14	14	0.023807	0.000398	0.001273	0.000359	0.000269
ko05152 Tuberculosis	6	8	10	9	/	0.025194	0.029589	0.002179	0.020763	
ko04610 Complement and coagulation cascades	4	6	/	/	/	0.035686	0.012969	/	/	/
ko05310 Asthma	2	/	/	/	/	0.04069	/	/	/	/
ko04260 Cardiac muscle contraction	/	9	10	8	8	/	0.001908	0.000315	0.016778	0.014398
ko00020 Citrate cycle (TCA cycle)	/	4	/	4	/	/	0.017886	/	0.027372	/
ko00280 Valine, leucine and isoleucine biosynthesis	/	2	/	2	/	/	0.020492	/	0.026165	/
ko03010 Ribosome	/	7	/	8	8	/	0.028186	/	0.016778	0.014398
ko00620 Pyruvate metabolism	/	4	/	/	/	/	0.028656	/	/	/
ko04612 Antigen processing and presentation	/	/	6	6	7	/	/	0.001748	0.00378	0.000409
ko04670 Leukocyte transendothelial migration	/	/	9	/	/	/	/	0.006172	/	/
ko03050 Proteasome	/	/	4	/	/	/	/	0.009282	/	/
ko04510 Focal adhesion	/	/	13	/	/	/	/	0.01106	/	/
ko05133 Pertussis	/	/	5	/	/	/	/	0.017658	/	/
ko00860 Porphyrin and chlorophyll metabolism	/	/	3	/	/	/	/	0.021891	/	/
ko05110 Vibrio cholerae infection	/	/	5	/	/	/	/	0.025512	/	/
ko05110 Pathogenic *Escherichia coli* infection	/	/	8	/	/	/	/	0.032446	/	/
ko05140 Leishmaniasis	/	/	5	/	/	/	/	0.0353	/	/
ko05322 Systemic lupus erythematosus	/	/	5	/	/	/	/	0.047123	/	/
ko05412 Arrhythmogenic right ventricular cardiomyopathy (ARVC)	/	/	6	/	/	/	/	0.049575	/	/
ko05134 Legionellosis	/	/	/	5	/	/	/	/	0.031754	/
ko00040 Pentose and glucuronateinterconversions	/	/	/	/	3	/	/	/	/	0.030341
ko00860 Porphyrin and chlorophyll metabolism	/	/	/	/	3	/	/	/	/	0.030341
ko00053 Ascorbate and aldarate metabolism	/	/	/	/	3	/	/		/	0.003884

**Fig. 5:**
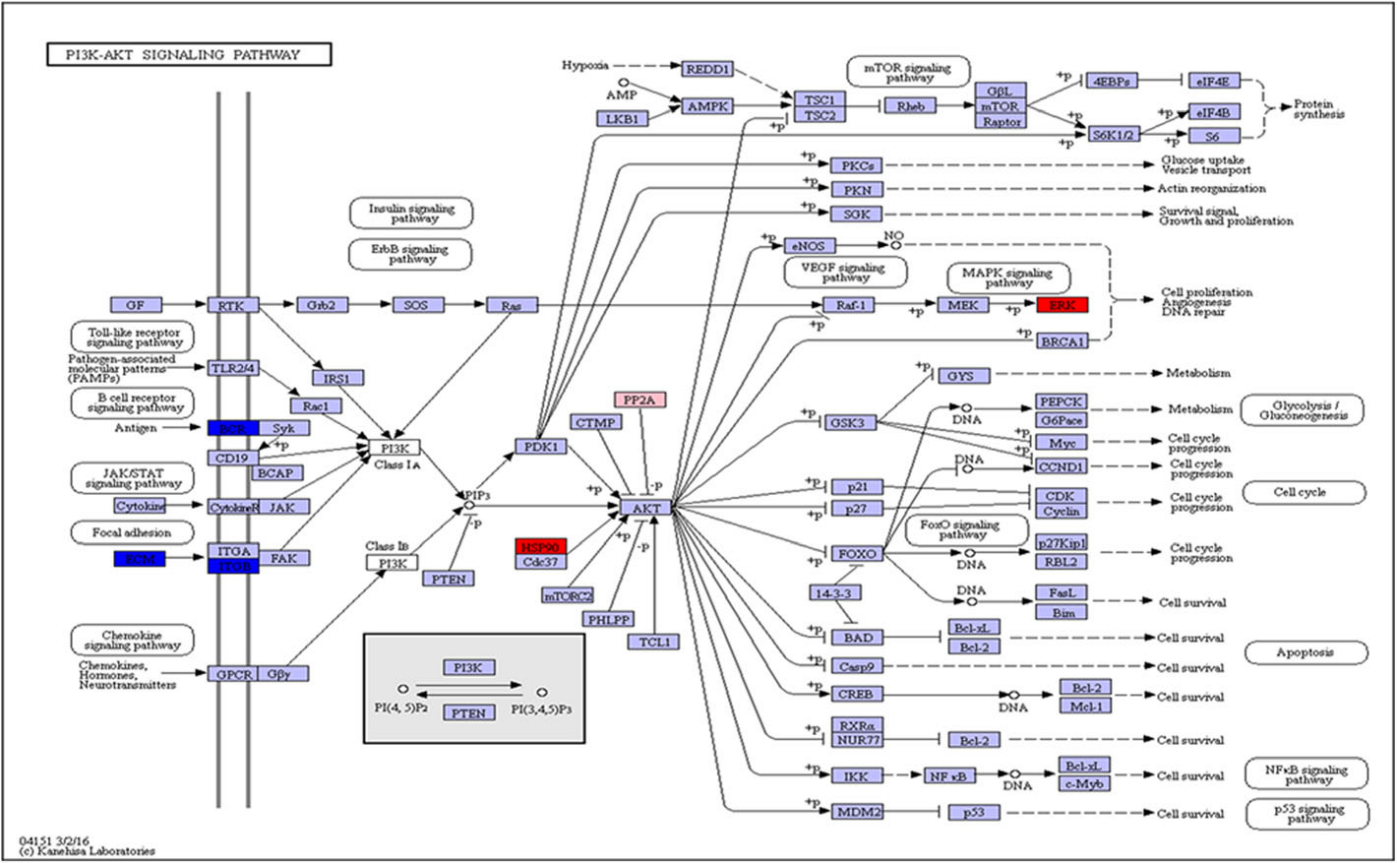
Protein expression mapped to the KEGG pathway. The protein expression profiles mapped to the PI3k-Akt signaling pathway. DEPs are marked with different colors. The red color indicates up-regulated, blue color indicates down-regulated, and pink indicates no obvious change

To illustrate changes in biological processes, protein expression data were mapped onto the KEGG Mapper-Search & Color Pathway (http://www.genome.jp/kegg/tool/map_pathway2.html) and changes in expression are indicated by color (Fig. 5). PI3k-Akt was the most enrichment pathway which associated with nine DEPs. IgA1 immunoglobulin, Collagen alpha-1(II) chain (COL2A1), Collagen alpha-3(VI) chain (CO6A3), Collagen alpha-1(XI) chain (COL11A1) and ITGB4 were down-regulated during regeneration, whereas heat shock protein HSP 90-alpha (HSP90AA1), Hemopexin (HPX), and Mitogen-activated protein kinase 3(MAPK3) were up-regulated. Serine/threonine-protein phosphatase2A regulatory subunit A (PPP2R1A) showed no obvious changes. Three genes (HPX, COL2A1 and ITGB4) involved in this pathway were randomly selected for qRT-PCR, the result was consistent with that of iTRAQ assay (Additional file 6: Figure S2).

### PPI network construction

The STRING database aims to provide a global perspective for as many organisms as is feasible. To investigate the interactions among regeneration-related proteins, DEPs predicatively related to wound healing and the immune response, cell proliferation and differentiation were integrated with information from the STRING database to construct a PPI network (Additional file 7: Table S5).

**Fig. 6:**
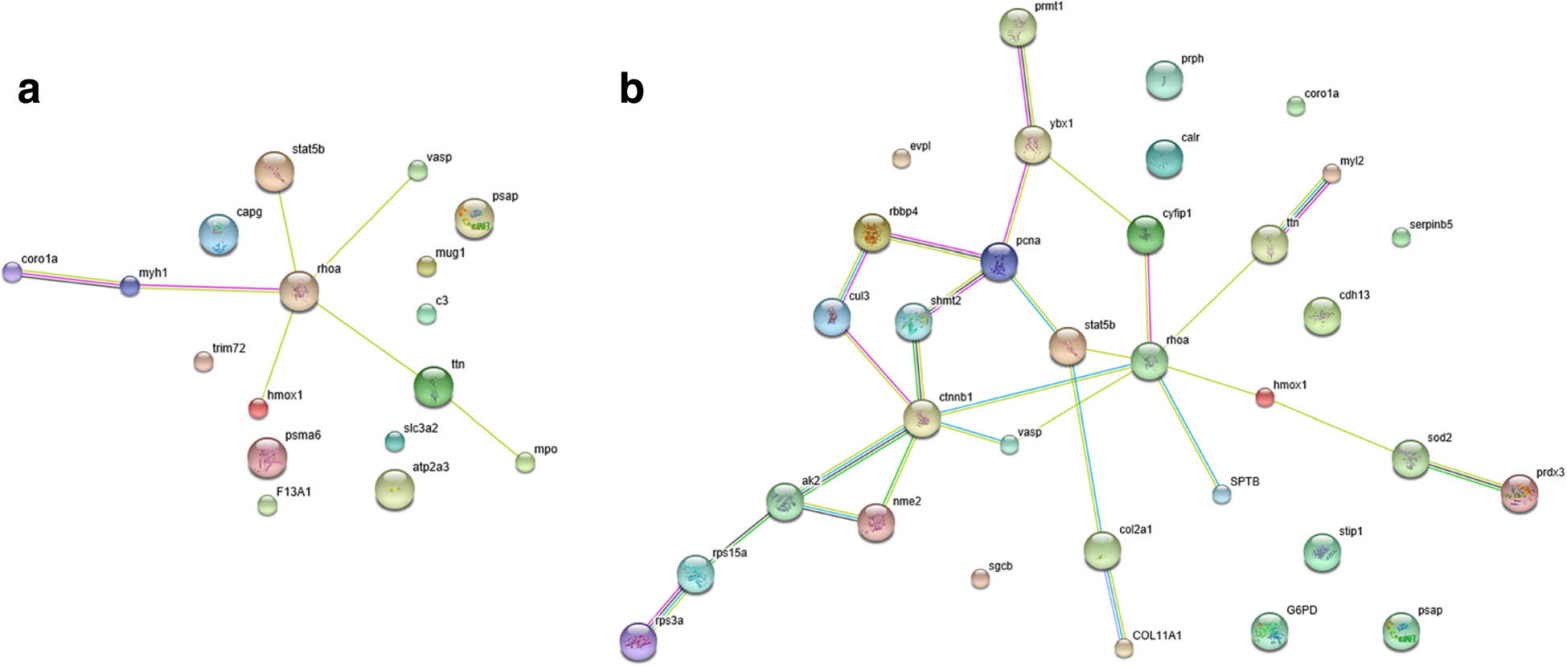
Interaction networks of wound healing and the immune response (**a**), cell proliferation and cell differentiation-related DEPs (**b**) identified by the iTRAQ experiment or Text Mining databases. The light blue lines represent data from curated databases. The purple lines represent experimentally determined data, and the yellow lines represent textmining. The black lines represent co-expression. The green lines represent the gene neighborhood, and the blue lines represent curated databases

A network was generated based on the protein–protein interactions of the 27 wound healing and immune response-related proteins in Fig. 6a. Only RHOA was located in the center of the network, indicating that proteins in the *Xenopus* Silurana database were limited.

CTNNB1 and RHOA were the key proteins for cell proliferation and cell differentiation in the PPI network. In Fig. 6b, an interaction network including STAT5, RHOA, PCNA, NME2, SHMT2, CTNNB1, HMOX1, VASP and CUL3 was generated, whereas no interactions were observed among CDH13, CALR, CORO1A, STIP, G6PD, Serpinb5 and EVPL.

### Validation of differentially expressed proteins by qRT-PCR analysis

**Fig. 7:**
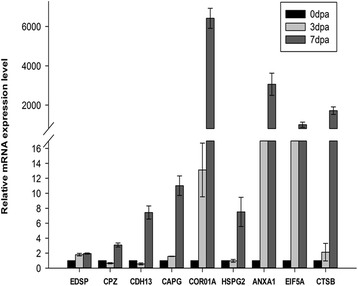
Quantitative RT-PCR analyses of gene expression in regenerating limb. Quantitative expression of genes for the regenerating time point calculated based on Ct value normalized against the housekeeping Beta-actin gene

To provide further information regarding the correspondence between proteins and their mRNA expression patterns, quantitative real-time polymerase chain reaction (qRT-PCR) was performed to investigate the dynamic transcriptional expression patterns of nine representative DEPs. The expression patterns determined by qRT-PCR were consistent with those obtained by iTRAQ, with 80% agreement between the qRT-PCR and iTRAQ results (Fig. 7). This result indicated that the differential proteomic analysis results in this study were reliable.

## Discussion

iTRAQ combined with LC–MS is one of the most powerful methodologies in quantitative proteomics [25, 26]. In the present study, we employed this technique to study protein expression profiles during limb regeneration to identify proteins that are critical for regeneration. Our findings provide primary proteomic information during limb regeneration that may be useful in understanding the mechanisms of limb regeneration. We detected a total of 6042 peptides and 2636 proteins in all samples (0, 3, 7, 14, 30 and 42dpa), whereas Rao et al. [21] detected 1624 peptides in all of their samples (0, 1, 4, and 7dpa). However, some proteins that were previously reported to be critical for limb regeneration were not identified in our study or Raos’ study, such as Fgf-8, Hox, sonic hedgehog, prod1 and nAG. This difference may due to an inability of the current LC-MS/MS technology to sensitively detect low abundance proteins.

The grouping of the proteins into clusters combined with functional classification analysis provided clear evidence for the involvement of different characteristic temporal patterns of protein expression during *Cynops orientalis* limb regeneration. Our results confirmed a number of earlier studies on signaling, cytoskeletal, ECM and metabolism changes.

### Wound healing stage

Fourteen DEPs for wound response and healing post amputation were detected in the current experiment (Additional file 7: Table S5), including RHOA, annexinA2, complement factor B, complement C3, coagulation factor XIII, and Signal transducer activator of transcription 5B (STAT5B). RHOA links plasma membrane receptors to the assembly of focal adhesions and actin stress fibers and plays an essential role for the apical junction formation of keratinocyte cell-cell adhesion. Previous study also validated the association of annexin A2 down-regulation for cell proliferation and differentiation in the mesenchymal progenitor cells of regenerating limbs [22].

Skin wounds in adult mammals heal after the re-epithelization on the formed granulation tissue and often result in scars or keloids. In salamander, the wound epithelium is formed quickly and then thickens to form the AEC. A featured histological appearance of AEC is the absence of the basal membrane, which enables the direct interaction among the epithelium, nerves, and mesenchymal tissue. Coincident with this histological appearance, the cell density was much higher at the amputation plane, indicating that the blastema forms primarily by the distal migration and accumulation of dedifferentiated cells under the wound epidermis [9]. Transplantation of full thickness skin graft onto the stump will terminate the regeneration process. In the current experiment, we detected the upregulation of Villin-1 and epidermal differentiation-specific protein, which could accelerate the formation of the wound epithelium. Moreover, the expression of basement membrane-specific heparan sulfate proteoglycan core protein, which is a major component of the basal membrane, was down-regulated.

The immune system is critical for the body’s defense against exogenous pathogens. Previous histological studies have indicated the presence of lymphocytes and macrophages in early blastema after limb amputation [21, 27]. In recent years, there has been a growing appreciation of the contribution of cellular components of the immune system to regeneration. Macrophages are professional phagocytes of the system. Regulation of the immune milieu has been suggested to be a critical precondition for adult regeneration. It was reported that systemic macrophage depletion in salamander may result in permanent failure of limb regeneration associated with extensive fibrosis and disregulation of extracellular matrix component gene expression [28]. In a recent study, Cattin and Burden et al. demonstrated that macrophages are necessary for the bridge of the migrating cords of Schwann cells during nerve damage [29]. We identified 16 proteins related to the immune response in the current study (Additional file 7: Table S5). Among them, 4 proteins were closely associated with macrophage function, including Coronin-1A, Macrophage-capping protein, Complement factor B and Complement C3. Coronin-1A, an actin binding protein that is involved in the regulation of the immune system [30, 31], was up-regulated at 7dpa and lasting until 42dpa. Macrophage-capping protein, a gelsolin family actin-regulatory protein involved in cell signaling, phagocytosis and motility [32], was significantly up-regulated at 3dpa and 7dpa. These results further implied the critical role of macrophages during limb regeneration. An in-depth understanding of the role of immune systems on regeneration may provide clues to therapeutic avenues to restore damaged tissue in mammals.

### Blastema formation

The major signal transduction pathways identified during regeneration included FGF, Wnt, Hedgehog (Hh), retinoic acid (RA), Notch, BMP, IGF, PI3K-AKT and MAPK signaling pathways. The Wnt pathway is essential for the proliferation and maintenance of stem cells, and is involved in the initial step of limbs morphogenesis and regeneration. Yokoyama H et al. demonstrated that inhibition of Wnt/β-catenin signaling could inhibit limb regeneration during the early stage [33]. In the current experiment, carboxypeptidaseZ (CPZ), a secreted Zn-dependent enzyme that is enriched in the extracellular matrix [34, 35], was up-regulated at 7, 14, 30 and 42dpa. MAPK3, which regulates various cellular processes such as cell proliferation, cycle progression, response to extracellular signals, was found up-regulated at 14dpa.

Nerves play a critical role in tissue repair and regeneration. In the case of salamander limb regeneration, regenerating axons are key elements and denervation may inhibit the ignition of blastemal formation. This effect is mediated by the interaction of nerves with the WE. In the current study, we detected two up-regulated proteins that are closely related to axon growth. Proactivatorpoly peptide (PSAP) is the precursor of sphingolipid activator proteins and is abundant in the nervous system and muscle, which supplied neurotrophic effects in vivo or in vitro [36]. RHOA is a member of the Rho family of small GTPases, which regulates cytoskeletal dynamics, transcription, cell cycle progression and cell transformation [37]. Peripheral nerve injury triggers the activation of RHOA in spinal motor and peripheral sensory neurons [38].

### Cell differentiation and limb regeneration

Cells adjacent to the amputation site were freed by protease-induced histolysis (Proteasome activator complex and Cathepsin B, as found in the current experiment) and dedifferentiated. Dedifferentiated cell were characterized by the expression of genes associated with the dedifferentiated state, including *msx1*, *Nrad*, *Notch*, *Klf4, Sox2, c-myc and LIN28* [15, 18, 39]*.* It has been suggested that apoptotic pathways are closely related to cell dedifferentiation [40]. In this study, nucleoside diphosphate kinase B, a member of the nm23 family and inhibitor of *c-myc* transcription, was found down-regulated.

Cell differentiation is crucial for limb regeneration, and cells in the blastema may undergo proliferation and differentiation to generate the missing structures of limb, such as skin, muscle, bone, cartilage, nerve and blood vessels. During this stage, regenerating tissue exhibited active biosynthetic activity, and 28 proteins were found associated with cell differentiation (Additional file 7: Table S5). In addition, 26 and 18 proteins involved in transcription and translation were detected (Additional file 7: Table S5). For example, calreticulin (CALR) was significantly up-regulated at 14 and 30dpa. Eukaryotic translation initiation factor 5A-2 was significantly up-regulated at all time points.

Analysis of the protein expression profile is useful to identify the initial molecules and mechanisms triggering limb regeneration and the difference in the wound healing pattern between salamander and mammals. Mimicking the mechanisms of limb regeneration in salamander may improve regenerative ability in humans via gene therapy, cell therapy and chemical therapy.

## Conclusions

In this study, an iTRAQ-based quantitative proteomic approach was applied to detect the overall proteome changes during *Cynops orientalis* limb regeneration. The results indicated that significant proteomic alternations occurred during the process. Totally 253 proteins were found differentially expressed and involved in 29 KEGG pathways. The DEPs detected may provide fundamental basis to understand the mechanism of limb regeneration. Several proteins, including coronin, RHOA and Macrophage-capping protein were first found differentially expressed, and the role of these proteins should be fully investigated in future study.

## Methods

### Sample preparation

*Cynops orientalis* were purchased from a pet market. All surgical procedures were performed under general anesthesia with 0.1% MS-222 (ethyl-p-aminobenzoate, Sigma-Aldrich, Steinheim, Germany) in a 1-L tap water-bath. The right forelimb of each animal was amputated 1 mm over elbow, and limb tissues were collected 3, 7, 14, 30 and 42dpa by cutting the arm 1 mm from the first amputation. The tissue removed distal to the amputation site acted as the control. The collected tissues were rinsed twice in filtered water and twice in 1× phosphate-buffered saline (PBS) and were then frozen in liquid nitrogen or stored at −80 °C until they were prepared for extraction.

### Histology

The tissues mentioned above were fixed in a PBS buffered formaldehyde solution for 48 h. Decalcification with EDTA was performed for 1 to 2 months. The fixed tissue samples were dehydrated in a graded series of alcohol, followed by xylene. Then, the samples were infiltrated overnight with paraffin. The tissues were then sectioned at 8 μm. Hematoxylin and eosin (HE) staining was performed.

### Protein extraction, iTRAQ labeling and SCX fractionation

Tissue samples from each group were ground into powder in liquid nitrogen, and a protein preparation was performed as described by Li et al. [41]. Three biological replicates from each group were used to prepare protein samples. The process of iTRAQ labeling and SCX fractionation was performed as described by Josselin et al. [25, 26]. Briefly, one hundred micrograms of protein sample was digested with Trypsin Gold (Promega, Madison, WI, USA) and labeled according to the manufacturer’s protocol for 8-plex iTRAQ reagent (Applied Biosystems). Samples were labeled with the iTRAQ tags as follow: control (113 tag), sample 3dpa (118 tag), sample 7dpa (121 tag), sample 14dpa (119 tag), sample 30dpa (117 tag), and sample 42dpa (115 tag). SCX chromatography was performed on a LC-20AB HPLC Pump system (Shimadzu, Kyoto, Japan) with a 4.6 × 250 mm^2^ Ultremex SCX analytical column. The labeled digests were subjected to LC-MS/MS analysis.

### LC-ESI-MS/MS analysis based on triple TOF 5600

Each fraction was resuspended in buffer A (5% ACN, 0.1%FA) and centrifuged at 20000 g for 10 min to achieve a final concentration of approximately 0.5 μg/μl. Then, 10 μl of supernatant was loaded on a LC-20 AD nanoHPLC (Shimadzu, Kyoto, Japan) by the auto sampler onto a 2-cm C18 trap column. Then, the peptides were eluted onto a 10-cm analytical C18 column packed in-house. The samples were loaded at 8 μL/min for 4 min. Then, the 35-min gradient was run as follows: 300 nL/min starting from 2 to 35% B (95%ACN, 0.1%FA); 5 min linear gradient to 60%, 2 min linear gradient to 80%, maintenance at 80% B for 4 min, and return to 5% in 1 min.

Data acquisition was performed with a TripleTOF 5600 System (AB SCIEX, Concord, ON) fitted with a Nanospray III source (AB SCIEX, Concord, ON), and a pulled quartz tip served as the emitter (New Objectives, Woburn, MA). Data were acquired using an ion spray voltage of 2.5 kV, curtain gas of 30 psi, nebulizer gas of 15 psi, and an interface heater temperature of 150. The MS was operated with a RP of greater than or equal to 30,000 FWHM for TOF MS scans. For IDA, survey scans were acquired over 250 ms, and up to 30 product ion scans were collected; a threshold of 120 counts per second (counts/s) was exceeded with a 2+ to 5+ charge-state. The total cycle time was fixed to 3.3 s. The Q2 transmission window was 100 Da for 100%. Four time bins were summed for each scan at a pulser frequency value of 11 kHz by monitoring the 40 GHz multichannel TDC detector with a four-anode channel to detect ions. A sweeping collision energy setting of 35 ± 5 eV coupled with iTRAQ adjust rolling collision energy was applied to all precursor ions for collision-induced dissociation. Dynamic exclusion was set at 1/2 of the peak width (15 s), and then, the precursor was refreshed off the exclusion list.

### iTRAQ data analysis

The MS data were processed by Proteome Discoverer software (version 1.2.0.208) (Thermo Scientific, Bremen, Germany), and protein identification and quantification were performed using the Mascot search engine (Matrix Science, London, UK; version 2.3.02) against the *Salamandridae* database containing 134,205 sequences.

For protein identification, a mass tolerance of 0.05 Da was permitted for intact peptide masses and 0.1 Da for fragmented ions, with an allowance for one missed cleavage in the trypsin digests. Gln- > pyro-Glu (N-term Q), oxidation (M), and deamination (NQ) were potential variable modifications. Carbamidomethyl (C), iTRAQ8plex (N-term), and iTRAQ 8plex (K) were fixed modifications. The charge states of peptides were set to +2 and +3. Specifically, an automatic decoy database search was performed in Mascot by choosing the decoy checkbox in which a random database sequence was generated and tested for raw spectra as well as the real database. To reduce the probability of false peptide identification, only peptides with significance scores (≥20) at the 99% confidence level with a Mascot probability analysis greater than “identity” were counted as identified. Each confident protein identification involved at least one unique peptide.

### Analysis of differentially expressed proteins

A protein must contain at least two unique peptides for protein quantitation. The quantitative protein ratios were weighted and normalized by the median ratio in Mascot. Only ratios with *p*-values <0.05 and fold changes of >1.2 or <0.8 were considered to be significant in the current study.

### Bioinformatics analysis

Functional annotations of the proteins were conducted with the Blast2GO program against the non-redundant protein database (NR; NCBI). The protein accessions list of native format containing corresponding GO annotations was submitted to the Web Gene Ontology Annotation Plot (WEGO: http://wego.genomics.org.cn/cgi-bin/wego/index.pl). The KEGG database (http://www.genome.jp/kegg/) and the COG database (http://www.ncbi.nlm.nih.gov/COG/)were used to classify and group these identified proteins.

To evaluate the similarities or differences of the protein expression profiles among different groups, Cluster3.0/TreeView software was used for hierarchical clustering of the identified proteins [42]. In the current study, the intensities of the identified proteins were used for hierarchical clustering, and the missing values were treated as zero.

### PPI network construction

STRING v10.1 (http://string-db.org/) was applied to analyze the protein–protein interaction (PPI) of differentially expressed protein identified in the current study and to construct PPI networks. The protein interaction information was extracted from the orthologous proteins of *Xenopus* Silurana. The active prediction methods were enabled, such as Database, Experiment and Text Mining [43].

### Quantitative real-time PCR (qRT-PCR) analysis for gene expression

qRT-PCR was performed using SYBR Premix Ex Taq (TaKaRa, Dalian, China) and a Thermal Cycler CFX96 Real Time-PCR detection system (Bio-Rad, Hercules, CA, USA) with the following parameters: 95 °C for 30 s; 40 cycles at 95 °C for 10 s; 52 °C for 10 s; and 72 °C 10 s. The β-actin gene was used as the internal control. For qRT-PCR, total RNA was extracted from the regenerating limb using the RNAiso plus reagent (Takara, Dalian, China) following the manufacturer’s protocols. The concentration and purity of total RNA were measured using a GE Nanovue™ Spectrophotometer (GE Healthcare Biosciences, Pittsburgh, USA). cDNA was synthesized using a SYBR Prime Script™ RT Master Mix (Perfect Real Time) Kit (Takara, Dalian, China). The gene-specific primers for qRT-PCR and gene annotation are listed in Table 2. The relative expression of each gene was calculated with the 2^−ΔΔCt^ method. There were three biological sample replicates, and each biological sample replicate included three technical replicates.

**Table 2:** Primers used in quantitative real-time PCR analysis

Target gene	Primer	Sequence (5’to3’)
Epidermal differentiation-specific protein	EDSP_F	GTGGAGCTGAAAATCGTCC
	EDSP_R	CATCAATGTAAGTTTCTGTGCC
Carboxypeptidase Z	CPZ_F	GATTAAGCCTGGTCGGGTAG
	CPZ_R	TGGGTCGAACTGATCTAACG
Cadherin-13	CDH13_F	AAGCCTTTGGACTACGAGA
	CDH13_R	ATGGACCTTCATTGACATCAC
Macrophage-capping protein	CAPG_F	TGACTGAACACGGGAAGG
	CAPG_R	CAATGCTGGAGATGACGC
Coronin-1A	CORO1A_F	GCTTTGATGAACCATTGACG
	CORO1A_R	TGTGACTCCTTGCTGCTGTAC
Basement membrane-specific heparan sulfate proteoglycan core protein	HSPG2_F	CAATACTGTGAGCGATGTGC
	HSPG2_R	ACTTGAACGATGAAAGGAGC
Annexin A1	ANXA1_F	TTACGAAACTATTCTGGTGGC
	ANXA1_R	TTTTGGGATTACAGGCACA
eukaryotic translation initiation factor 5A isoform II	EIF5A2_F	AAGATACGAGGCAGGAGAAG
	EIF5A2_R	AAGTGGGCGATTATTTGGT
Cathepsin B	CTSB_F	CTGTCTTGCTGTGGTCTGG
	CTSB_R	AGGTTGTCACGCATTTGG
Beta-actin	ACTB_F	CTCAACCCCAAAGCCAATC
	ACTB_R	GAAGCATACAGGGACAGCA
Hemopexin	HPX_F	CGCTCGGATCAGCTCTACAC
	HPX_R	TTCAGGTCCACCAAAGAAAGG
Integrin beta-4	ITGB4_F	TTCGCTTGACCAATGATGTAGA
	ITGB4_R	GGCTTCGTAGTGAAATGCTGAC
Collagen alpha-1(II) chain	COL2A1_F	CCTGGAACACCTGGCACTG
	COL2A1_R	TCACCTACGTCACCCCTGTCT

## Additional files

Additional file 1: Table S1. Details for the identified proteins. (XLS 3255 kb)

Additional file 2: Table S2. The differentially expression proteins (DEPs) identified during *Cynops orientalis* limb regeneration. (XLSX 194 kb)

Additional file 3: Figure S1. Hierarchy clustering analysis of the DEPs on the limb regenerated stages of *Cynops orientalis*. (ZIP 670 kb)

Additional file 4: Table S3. GO annotations of the differentially expressed proteins. (XLSX 20 kb)

Additional file 5: Table S4. Differential expression proteins were assigned to 232 KEGG pathway. (XLSX 22 kb)

Additional file 6: Figure S2. qRT-PCR validation of three selected genes involved in PI3k-Akt signaling pathway. (TIFF 122 kb)

Additional file 7: Table S5. Gene ontology function enrichment analysis of DEPs involved in PPI network construction. (XLSX 15 kb)

## Abbreviations

MAPK3: Mitogen-activated protein kinase 3
3PPP2R1A: Serine/threonine-protein phosphatase 2A 65 kDa regulatory subunit A alpha isoform
CALR: Calreticulin
CAPG: Macrophage-capping protein
CDH13: Cadherin-13
COL2A: Collagen alpha-1(II) chain
CORO1A: Coronin-1A
CTNNB1: Catenin beta-1
CUL3: Cullin-3
DEPs: Differentially expressed proteins
DPA: Days post amputation
EVPL: Envoplakin
FDR: False discovery rate
G6PD: Glucose-6-phosphate 1-dehydrogenase
GO: Gene Ontology
HMOX1: Heme oxygenase 1
HPX: Hemopexin
HSP90AA1: Heat shock protein HSP 90-alpha
ITGB4: Integrin beta-4
iTRAQ: isobaric tags for relative and absolute quantification
KEGG: Kyoto Encyclopedia of Genes and Genomes
LC-MS/MS: liquid chromatography tandem mass spectrometry
NME2: Nucleoside diphosphate kinase B
PCNA: Proliferating cell nuclear antigen
RHOA: Transforming protein RhoA
SHMT2: Serine hydroxymethyltransferase,mitochondrial
STAT5: Signal transducer and activator of transcription 5B
STIP: Stress-induced-phosphoprotein 1
VASP: Vasodilator-stimulated phosphoprotein
