# Supplementary figures and images for: ITRAQ-based quantitative proteomic analysis of Cynops orientalis limb regeneration
Source: BMC Genomics. 2017 Sep 22;18:750. doi: 10.1186/s12864-017-4125-4 (PMC5610437; doi:10.1186/s12864-017-4125-4)

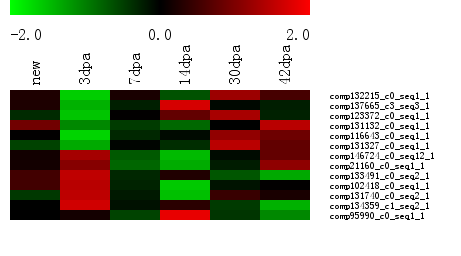

Supplement: Supplementary file 3 — Hierarchy clustering analysis of the DEPs on the limb regenerated stages of Cynops orientalis. (ZIP 670 kb) [file 12864_2017_4125_MOESM3_ESM.zip › Supplement Figure 1C.tif]

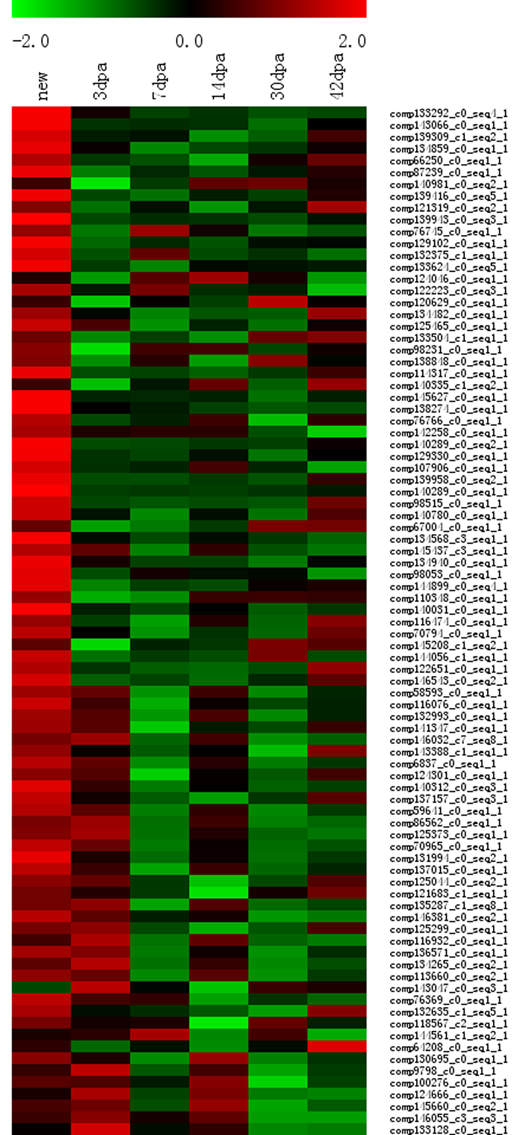

Supplement: Supplementary file 3 — Hierarchy clustering analysis of the DEPs on the limb regenerated stages of Cynops orientalis. (ZIP 670 kb) [file 12864_2017_4125_MOESM3_ESM.zip › Supplement Figure 1B.tif]

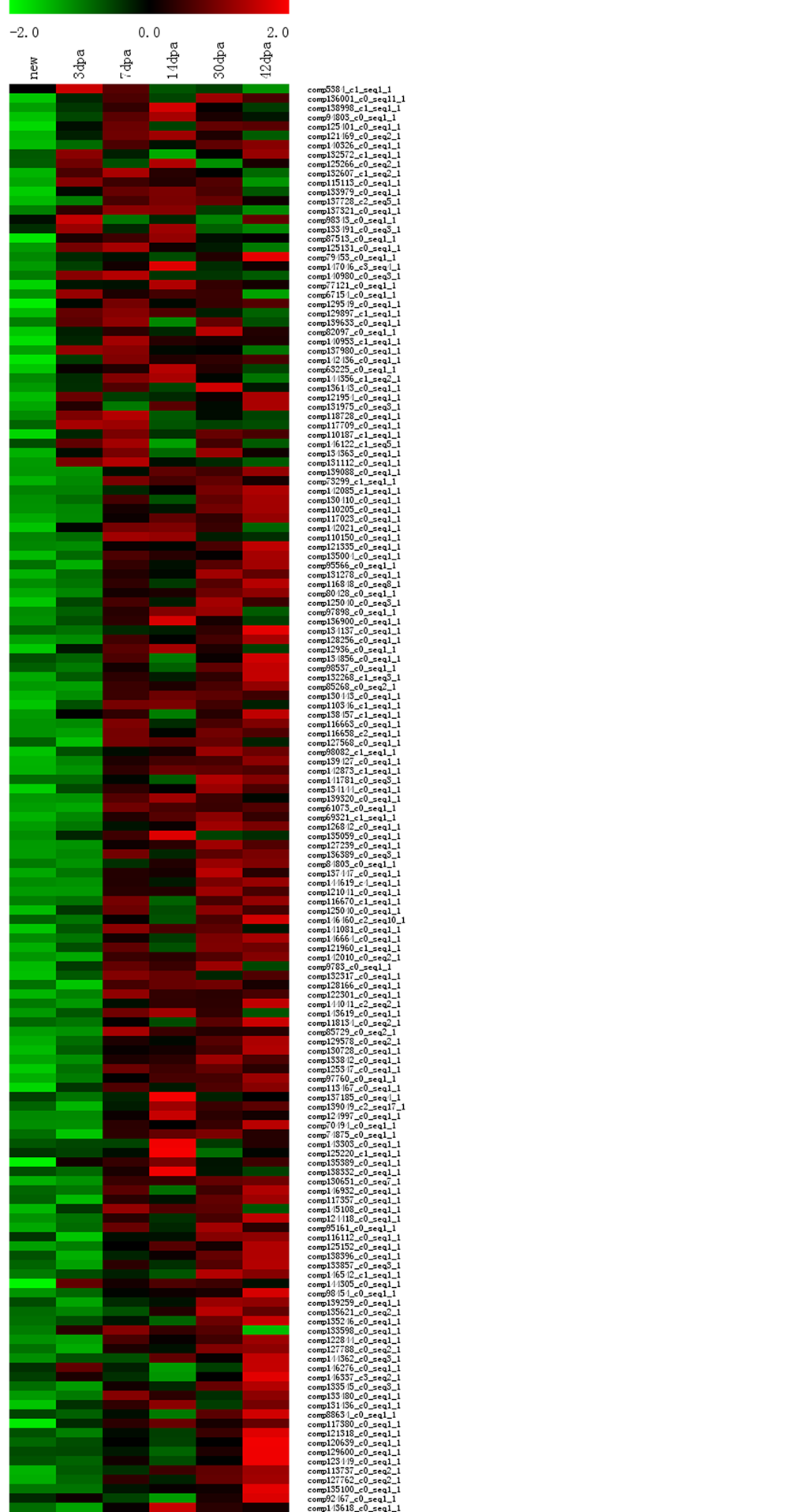

Supplement: Supplementary file 3 — Hierarchy clustering analysis of the DEPs on the limb regenerated stages of Cynops orientalis. (ZIP 670 kb) [file 12864_2017_4125_MOESM3_ESM.zip › Supplement Figure 1A.tif]

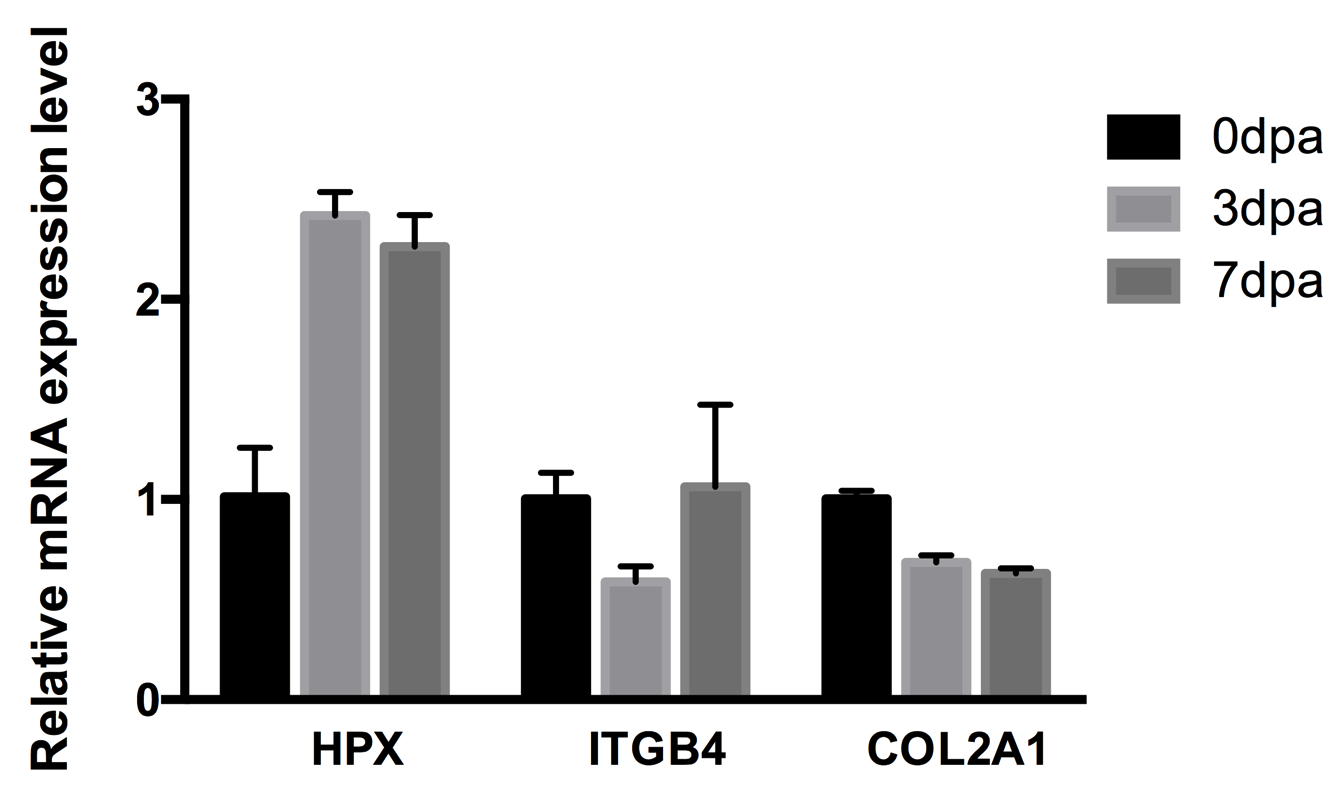

Supplement: Supplementary file 6 — qRT-PCR validation of three selected genes involved in PI3k-Akt signaling pathway. (TIFF 122 kb) [file 12864_2017_4125_MOESM6_ESM.tiff]
